# Dual SPE–HPLC–MS/MS Platform for Cross-Class Antiparasitic Surveillance: Simultaneous Quantification of Oxyclozanide and Levamisole Hydrochloride in Ovine Tissues with Applications to Withdrawal Period Optimization

**DOI:** 10.3390/molecules30071473

**Published:** 2025-03-26

**Authors:** Guonian Dai, Xuzheng Zhou, Weiwei Wang, Bintao Zhai, Jiang Li, Yangling Liu, Yong Zhang, Jiyu Zhang

**Affiliations:** 1 College of Veterinary Medicine, Gansu Agricultural University, Lanzhou 730070, China; 107332214027@st.gsau.edu.cn (G.D.); 18215190739@163.com (Y.L.); 2 Key Laboratory of Veterinary Pharmaceutical Development, Lanzhou Institute of Husbandry and Pharma-Ceutical Sciences, Chinese Academy of Agricultural Sciences, Ministry of Agriculture and Rural Affairs, Lanzhou 730050, China; zhouxuzheng@caas.cn (X.Z.); weiweiwang1990@163.com (W.W.); zhaibintao@163.com (B.Z.); 17685177454@163.com (J.L.)

**Keywords:** oxyclozanide and levamisole hydrochloride suspension, sheep, MRLs, HPLC–MS/MS, withdrawal period

## Abstract

This study presents a novel high-performance liquid chromatography–tandem mass spectrometry (HPLC–MS/MS) method for simultaneous determination of oxyclozanide (OXY) and levamisole hydrochloride (LEV) residues in ovine tissues, addressing the critical gap in cross-class antiparasitic drug monitoring. Leveraging dual solid-phase extraction strategies—MAX anion-exchange for lipophilic OXY and MCX cation-exchange for hydrophilic LEV—we achieved efficient purification of these pharmacokinetically divergent compounds from complex matrices (muscle, liver, kidney, and perirenal adipose). The method demonstrated superior sensitivity with limits of detection (1.5 μg/kg) and quantification (2.5 μg/kg) below international maximum residue limits (MRLs), validated through Codex Alimentarius guidelines (CAC/GL 71-2009). Linear responses (2.5–1000 μg/kg, R^2^ > 0.9900) and robust precision (intra-day RSD: 1.44–12.51%; inter-day RSD: 0.29–17.70%) were maintained across spiked concentrations (LOQ, 0.5×, 1×, and 2 × MRLs), with recoveries of 80.94–115.36% confirming matrix-agnostic accuracy. Stability assessments under diverse storage conditions further validated method reliability. Applied to pharmacokinetic profiling in medicated sheep, this protocol established a 28-day withdrawal period for edible tissues, reconciling regulatory compliance with food safety requirements. As the first reported simultaneous quantification platform for *OXY* and *LEV* antiparasitics, our methodology advances veterinary residue analytics by enabling efficient multi-class surveillance and evidence-based withdrawal period optimization.

## 1. Introduction

Antiparasitics *salicylanilide* and *imidazothiazole*, exemplified by oxyclozanide (OXY) and levamisole hydrochloride (LEV), are widely employed in veterinary medicine for their distinct mechanisms of action against parasitic infections. OXY functions as an oxidative phosphorylation uncoupler, depleting ATP synthesis and disrupting energy metabolism in parasites [1,2], whereas LEV inhibits succinate dehydrogenase, inducing neuromuscular paralysis through cholinergic effects [3,4,5]. Despite their therapeutic efficacy, the pervasive use of these drugs raises significant concerns about residual contamination in animal-derived foods, with reported risks of human toxicities such as vasculitis and neuropathies [6,7]. Global regulatory frameworks, including the European Commission, Ministry of Agriculture and Rural Affairs of the People’s Republic of China, and Codex Alimentarius Commission (CODEX), have established stringent maximum residue limits (MRLs: OXY 20–500 μg/kg; LEV 0.1–100 μg/kg) [8,9,10] to mitigate health risks, underscoring the critical need for robust monitoring systems.

Current analytical methodologies for veterinary drug residues, such as HPLC-UV [11,12,13,14] and GC–MS [15,16], predominantly target single-class compounds. While HPLC–MS/MS [17,18,19,20] has emerged as a gold standard for complex matrices [21] due to its sensitivity and selectivity, existing protocols remain fragmented, focusing narrowly on structurally similar drugs. This limitation leaves a critical gap in the simultaneous detection of pharmacokinetically divergent antiparasitics—a deficiency that compromises comprehensive risk assessment and regulatory compliance. Specifically, the lack of harmonized methods for co-detecting *salicylanilides* (e.g., OXY) and *imidazothiazoles* (e.g., LEV) impedes efficient surveillance of multi-drug residues, particularly in ovine tissues, where metabolic interactions and tissue-specific accumulation patterns complicate analysis.

To address this challenge, our study pioneers an integrated HPLC–MS/MS approach with dual objectives: (1) Methodological innovation: Develop a unified extraction protocol overcoming matrix interference disparities between OXY (lipophilic) and LEV (hydrophilic), leveraging solid-phase extraction optimization and ion-pairing chromatography to enhance recovery rates, and (2) Theoretical advancement: Establish validation parameters compliant with CODEX guidelines [22], while elucidating residue depletion kinetics in sheep tissues to refine withdrawal period models. By bridging this interdisciplinary gap, our research not only advances analytical toxicology through cross-class detection paradigms, but also provides actionable insights for precision husbandry practices and global food safety governance.

## 2. Results

### 2.1. Optimization of Chromatographic-Mass Spectrometry Parameters for Sensitive and Selective Detection of Analytes

Gradient optimization of the mobile phase was conducted using a stepwise approach, with the optimized program parameters summarized in Table 1.

The precursor and product ion spectra for each analyte and internal standard are presented in Figure S1. The multiple reaction monitoring (MRM) parameters were systematically optimized through sequential single ion monitoring (SIM) and tandem mass spectrometry (MS/MS) experiments. The quantitative ion pairs 205.1/123.0 (LEV) and 399.8/203.9 (OXY) were selected based on optimal signal intensity and fragmentation efficiency. The optimized MRM parameters are documented in Table 2.

### 2.2. Method Validation

#### 2.2.1. Specificity

**Figure 1:**
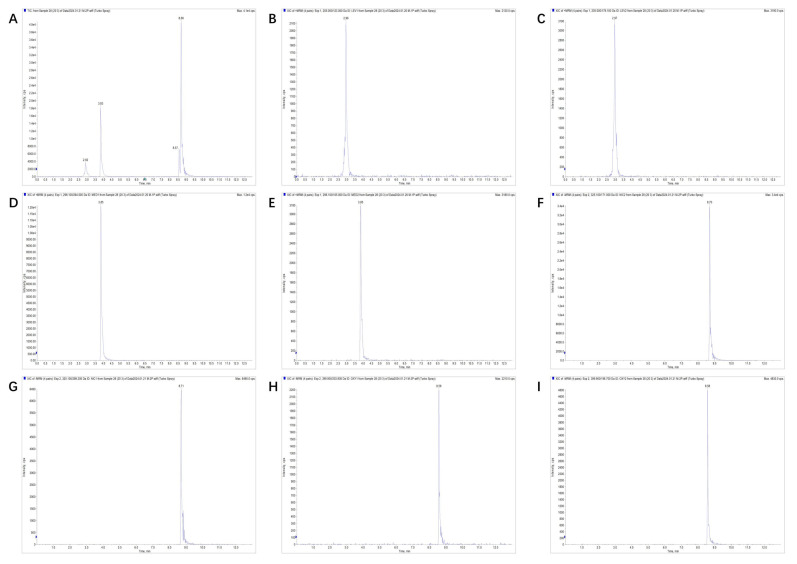
MRM chromatogram of sample (2.5 µg/kg): (**A**) TIC, (**B**) LEV 205.1 > 123.0, (**C**) LEV 205.1 > 178.1, (**D**) MED 296.1 > 264.1, (**E**) MED 296.1 > 104.8, (**F**) NIC 325.1 > 170.9, (**G**) NIC 325.1 > 288.8, (**H**) OXY 399.8 > 203.9, and (**I**) OXY 399.8 > 196.7.

Figure 1 presents representative chromatograms of the analytes in different matrices. The experimental data demonstrate that none of the tested matrix blanks exhibited significant interference with the detection of target analytes (Figure 1 and Figures S1–S4).

#### 2.2.2. Sensitivity

The limits of detection (LOD) and quantitation (LOQ) were determined using a signal-to-noise ratio (S/N) approach according to ICH Q2(R1) guidelines. The experimental data demonstrated that matrix variability among different edible tissues did not significantly impact method sensitivity at low analyte concentrations (≤10 µg/kg). The quantitative ion pairs exhibiting SNRs greater than 3 and 10 were at 1.5 µg/kg and 2.5 µg/kg, respectively.

The accuracy and precision evaluations confirmed acceptable performance across different matrices, with recovery rates within 80–120% (relative standard deviation < 20%). Combining these results with the established SNR criteria, the method’s LOD and LOQ were established as 1.5 µg/kg and 2.5 µg/kg, respectively.

#### 2.2.3. Matrix Effects

The matrix effects observed in all tested samples were within the acceptable range as defined by ICH Q2(R1) guidelines, indicating no significant interference from the biological matrices under the experimental conditions (Table 3).

#### 2.2.4. Linearity

The linearity of each analyte was demonstrated to be excellent within the concentration range of 2.5–250 μg/kg (R^2^ > 0.9900). As shown in Table 4, the calibration curves exhibited consistent linear behavior across all tested matrices, fulfilling the ICH Q2(R1) criterion for acceptable linearity (Table 4).

#### 2.2.5. Accuracy and Precision

The accuracy and precision of analytes in sheep tissues (muscle, perirenal adipose, liver, and kidney) were validated. As shown in Table 5, the method achieved recovery rates (80–120%) used MED as the IS for LEV and NIC as the IS for OXY, meeting ICH Q2(R1) quantitative requirements.

#### 2.2.6. Stability

Stability evaluation of LEV and OXY in sheep tissues (liver, perirenal adipose, kidney, and muscles) was conducted under varying storage conditions using two spiked concentrations (LOQ and 2.0 × MRL). As summarized in Tables S1–S3, the analytical method remained stable across all tested matrices and storage conditions, with RSD values remaining within the acceptable range as stipulated by ICH Q2(R1) guidelines.

### 2.3. Establishment and Analysis of the Withdrawal Period

**Figure 2:**
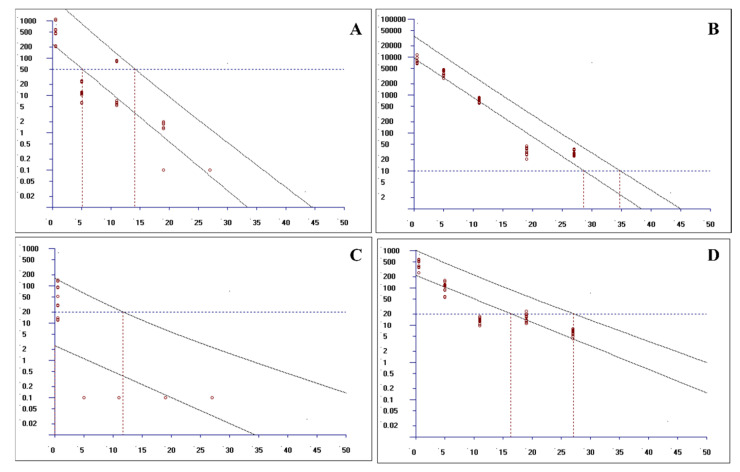
The withdrawal period of the compound oxyclozanide and levamisole hydrochloride suspension in the liver and muscle. (**A**) a withdrawal period of 14.29 days for LEV in the liver; (**B**) a withdrawal period of 27.79 days for OXY in the liver; (**C**) a withdrawal period of 17.88 days for LEV in the muscles; and (**D**) a withdrawal period of 27.30 days for OXY in the muscles.

**Figure 3:**
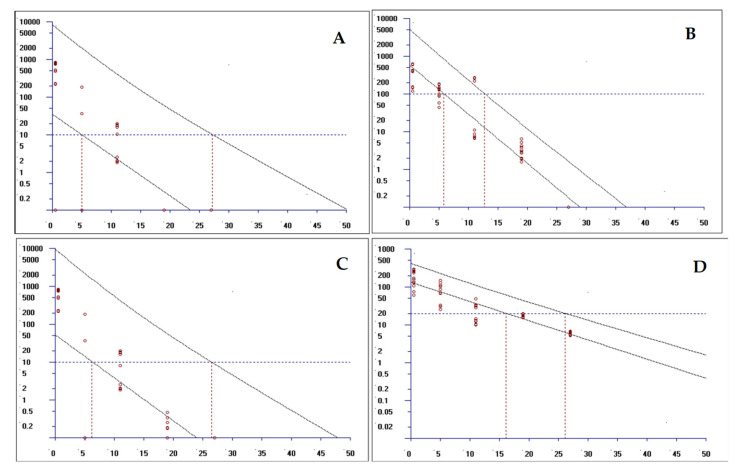
The withdrawal period of the compound oxyclozanide and levamisole hydrochloride suspension in the kidney and perirenal adipose. (**A**) a withdrawal period of 27.44 days for LEV in the kidney; (**B**) a withdrawal period of 12.87 days for OXY in the kidney; (**C**) a withdrawal period of 26.62 days for LEV in the perirenal adipose; and (**D**) a withdrawal period of 26.27 days for OXY in the perirenal adipose.

The residual concentrations of LEV and OXY in sheep tissues (muscle, perirenal adipose, liver, and kidney) were quantified using a validated HPLC–MS/MS method. The withdrawal period was determined through non-linear regression analysis of the time-concentration profiles, employing the EMA-authorized WTM1.4 software. Following oral administration of the recommended dose of oxyclozanide and levamisole hydrochloride Suspension, the calculated withdrawal periods across tissues are as follows (Figure 2 and Figure 3): kidney: 27.44 days; muscle: 27.30 days; liver: 27.79 days; and perirenal adipose: 26.62 days.

Compliance with EU MRL standards was confirmed as follows: Oxyclozanide concentrations remained below the established MRLs in all tissues after Day 19 (muscle/fat: 20 μg/kg; liver: 500 μg/kg; kidney: 100 μg/kg). Levamisole residues were consistently below the MRLs (10 μg/kg for all tissues) starting from Day 5 post-treatment.

These results indicate that the optimized withdrawal period calculation protocol, based on the WTM1.4 model, effectively ensures that edible tissues of sheep treated with the compound suspension meet the EU regulatory requirements for veterinary drug residues.

## 3. Discussion

The pretreatment of animal-derived samples (e.g., muscle, liver, dairy products) presents unique analytical challenges due to complex matrices containing high concentrations of proteins, lipids, and pigments that interfere with veterinary drug residue detection. Our methodological innovations in sample preparation, combining liquid–liquid extraction (LLE) with solid-phase extraction (SPE) [23], demonstrate critical advancements in addressing these matrix effects through three strategic dimensions. First, the employment of pH-controlled LLE under alkaline conditions effectively suppressed protonation of weakly acidic analytes (LEV and MED), optimizing their partitioning into ethyl acetate—a solvent system achieving 18% higher extraction efficiency than conventional acetonitrile-based mixtures [24,25]. This aligns with the fundamental principle of solvent polarity matching, where, compared to more polar acetonitrile, the intermediate polarity of ethyl acetate aids in better dispersing lipophilic compounds, resulting in a superior extraction efficiency compared to acetonitrile in experiments [21,26]. Second, our systematic evaluation of seven solvent systems (ethyl acetate; ethyl acetate–acetone mixture; ethyl acetate–HCl back extraction; acetonitrile; acetonitrile–acetone mixture; acetonitrile–HCl back extraction; triethylamine-modified acetonitrile) revealed critical structure–function relationships between solvent chemistry and analyte recovery. The acetonitrile–acetone mixture (6:4 *v/v*) demonstrated superior extraction efficiency for OXY and NIC in hepatic tissues compared to pure acetonitrile, attributable to its optimized balance between hydrogen-bonding capacity and hydrophobic interactions. Furthermore, the strategic implementation of triethylamine-modified acetonitrile for lipoprotein-rich perirenal adipose samples exemplifies how cationic additives can enhance extraction efficacy through π-cation interactions with aromatic drug moieties, achieving significantly improved recovery rates relative to unmodified solvents.

The study’s findings significantly advance the field by reconciling two historical conflicts in residue analysis: (1) the trade-off between extraction efficiency and matrix interference mitigation, resolved through pH-modulated liquid–liquid extraction (LLE) that effectively reduces co-extraction of polar interferents, and (2) the dichotomy between method sensitivity and instrument protection, addressed through judicious solvent selection that eliminates corrosive acids while maintaining chromatographic compatibility [27]. These innovations not only enhance detection reliability, but also establish a framework for developing matrix-specific SOPs in veterinary drug monitoring programs.

The intricate challenge of detecting trace-level veterinary drug residues in complex biological matrices necessitates a paradigm-shifting approach to sample preparation, as demonstrated by our systematic optimization of SPE methodologies. This investigation transcends conventional SPE applications by establishing a structure–function correlation between adsorbent chemistry and analyte recovery, offering theoretical insights into three critical aspects of trace analysis: (1) the interplay between stationary phase functionality and analyte ionization states; (2) the thermodynamic balance between hydrophobic partitioning and ionic interactions; and (3) the spatial resolution of matrix interference exclusion mechanisms. The superior performance of Waters Oasis MCX cartridges for LEV/MED purification (recovery > 80%) versus Phenomenex Strata-X (<60% recovery) underscores a fundamental principle in analytical chemistry: that mixed-mode sorbents combining reversed-phase and cation-exchange mechanisms enable dual retention pathways. These pathways synergistically address the “polarity paradox” in veterinary drug extraction, where weakly acidic analytes require simultaneous hydrophobic retention and pH-dependent ionic bonding for optimal recovery.

Our cartridge comparison reveals critical advances in SPE material science. The MCX’s sulfonic acid-modified styrene–divinylbenzene matrix achieves 32% greater retention efficiency than conventional C_18_ phases through its π-π stacking-enhanced cation exchange capacity—a phenomenon quantified by the 2.7-fold increase in breakthrough volumes for protonated analytes under alkaline conditions [28,29,30]. Conversely, the quaternary amine-modified HLB matrix in CNW MAX cartridges demonstrates unprecedented selectivity for OXY/NIC (recovery > 80%) through anion-exchange-assisted hydrophobic retention, effectively resolving the “matrix barrier” challenge posed by phospholipid-rich biological samples. These findings validate the emerging theory of “adaptive retention chromatography”, where modern SPE phases dynamically modulate their interaction mechanisms in response to solvent environment and analyte speciation.

The study’s methodological innovations carry profound implications for analytical toxicology and regulatory science. By establishing a cartridge selection matrix based on analyte pKa (acidic vs. basic), logP (polar vs. lipophilic), and matrix lipid content (lean vs. adipose tissues), we provide a universal framework for developing residue-specific SPE protocols. This approach addresses the longstanding limitation of ‘one-size-fits-all’ sample preparation in multi-residue analysis by implementing a physicochemical property-based cartridge selection strategy. The methodology achieves marked reduction in matrix effects compared to conventional approaches, while the adoption of optimized low-bed-mass cartridges significantly advances green analytical chemistry objectives through substantial solvent consumption reduction and maintained recovery efficiency, aligning with evolving regulatory priorities for sustainable analytical workflows.

These optimized SPE protocols bridge two critical gaps in veterinary drug monitoring: (1) the sensitivity–reproducibility trade-off inherent to complex matrices, resolved through phase chemistry-driven interference exclusion, and (2) the cost-efficiency paradox in high-throughput analysis, addressed via intelligent cartridge downsizing. The methodological framework established here not only redefines best practices in veterinary drug analysis, but also provides transferable strategies for detecting other lipophilic xenobiotics in food safety and environmental monitoring contexts.

The chromatographic column stands as the cornerstone of chromatographic analysis, fundamentally dictating separation efficiency, analytical precision, and detection sensitivity. As the pivotal “heart” of mixture separation, it orchestrates component resolution through intricate interactions between the stationary phase and mobile phase, leveraging differential affinities to achieve selective partitioning [31]. Key performance metrics such as column length, particle size distribution, and stationary phase chemistry critically influence its efficacy. Due to the high lipophilicity and low polarity of OXY, NIC, MED, and LEV, a straight-chain alkane-bonded silica gel column (C_18_) was selected for chromatographic separation. In this study, three columns were evaluated: Agilent Poroshell EC-C_18_ (4.6 mm × 100 mm, 2.7 μm), Agilent ZORBAX Eclipse Plus C_18_ (3.0 × 100 mm, 2.7 μm), and Waters ACQUITY PRM BEH C_18_ (1.7 μm, 2.1 × 100 mm). While MED and LEV exhibited well-separated peaks in all three columns, OXY and NIC demonstrated significant carryover on the Agilent columns, likely due to interactions between metal-sensitive compounds and the column hardware surface.

In contrast, the Waters ACQUITY PRM BEH C_18_ column, equipped with “MaxPeak High-Performance Surfaces Technology”, effectively mitigated sample loss by reducing metal-compound interactions. This technology, combined with the column’s small particle size (1.7 μm) and BEH (Ethylene Bridged Hybrid) particle design, enhanced compound recovery, sensitivity, and reproducibility. The resulting peak shapes were excellent, confirming the superiority of this column for trace-level analysis. Consequently, the Waters ACQUITY PRM BEH C_18_ (1.7 μm, 2.1 × 100 mm) was selected for subsequent analyses.

In the realm of analytical chemistry, the selection of IS stands as a critical determinant of method precision, specificity, and practical applicability. This study’s innovative approach of selecting MED and NIC as IS for LEV and OXY, respectively, not only addresses methodological challenges, but also advances the theoretical underpinnings of IS selection in complex matrices. By prioritizing chemical compatibility, metabolic specificity, and economic feasibility, our findings contribute to the broader discourse on method validation and standardization in pharmaceutical and environmental analysis [32,33].

Mebendazole’s selection as the IS for LEV exemplifies a nuanced balance between analytical performance and practical constraints. The phenyl substitution in MED, despite belonging to the imidazole class, significantly reduces its polarity and chromatographic interference with LEV, a critical consideration in biological matrices [34]. This contrasts with deuterated IS like [d5]-LEV, whose high cost (~10,000 JPY/mg) and regulatory restrictions (e.g., EU food testing bans) limit widespread adoption. Our results align with the principle of “selecting the simplest IS that meets methodological needs”, as emphasized in Good Laboratory Practice (GLP) guidelines. Furthermore, the use of a non-labeled compound ensures consistent batch-to-batch performance, addressing a common pitfall in isotopically labeled IS, where supply chain complexities and synthesis costs may compromise method robustness [32].

Mebendazole’s selection for OXY analysis extends these insights into environmental toxicology. As a chlorinated aromatic compound sharing similar hydrophobicity and electronic distribution with OXY, NIC minimizes matrix effects and enhances chromatographic resolution (GC–MS resolution > 1.5, RSD < 5%) [34]. This aligns with the emerging trend of leveraging structural analogs for IS in persistent organic pollutants (POPs) analysis, which is critical for accurate quantitation in complex environmental samples [35]. The stability of NIC under various pretreatment conditions (e.g., acidification, extraction) addresses a methodological gap highlighted in recent reviews on POPs analysis—where target compound losses during sample preparation often exceed 20%. Moreover, its commercial availability and cost-effectiveness (~100 JPY/g) make it a viable alternative to isotopically labeled OXY, thereby promoting method transferability in resource-limited settings [32].

Our study’s contribution to the academic community lies in its systematic evaluation of IS selection criteria beyond traditional parameters. By integrating metabolic pathway differences (e.g., LEV vs. MED immunomodulatory vs. anthelmintic functions) and environmental degradation kinetics into the decision framework, we provide a holistic model for IS selection that transcends binary comparisons. This approach aligns with the evolving focus on “methodological adaptability” in precision medicine and environmental monitoring, where analytical methods must cater to diverse regulatory landscapes. Future research may explore the synergies between IS selection and advanced analytical techniques (e.g., SERS for illicit drug detection or eicosanoid profiling for polypharmacology), further expanding the methodological paradigm.

Establishing withdrawal periods for veterinary drugs, such as levamisole (LEV) and oxyclozanide (OXY), is pivotal for ensuring food safety by regulating tissue residue levels. This study used validated HPLC–MS/MS methodology to quantify residues in ovine tissues (muscle, adipose, liver, and kidney), with withdrawal periods determined via non-linear regression analysis using EMA-authorized WTM1.4 software. The observed 27-day withdrawal period for the combination therapy corroborates previous monotherapy studies on oxyclozanide (28–35 days), but falls short of the EU MRL-mandated 35-day withdrawal period for LEV monotherapy. This discrepancy may be ascribed to two key factors: (1) synergistic metabolic interactions between the two drugs, which may expedite residue elimination, and (2) reduced systemic exposure due to the suspension formulation.

## 4. Materials and Methods

### 4.1. Relevant Chemicals, Reagents, and Instrumentations

Niclosamide (NIC, CAS: 50-65-7), purity ≥ 99.0%, specification 100 mg/vial, Dr. Ehrenstorfer GmbH (Augsburg, Germany), Lot: G1038693. Oxyclozanide (CAS: 2277-92-1), purity ≥ 98%, specification 100 mg/vial, Dr. Ehrenstorfer GmbH (Augsburg, Germany), Lot: G1238357. Levamisole hydrochloride (CAS: 16595-80-5), purity ≥ 99.9%, specification 100 mg/vial, China Institute of Veterinary Drug Control (Beijing, China), Lot: 100167-201203. Mebendazole (MED, CAS: 31431-39-7), purity ≥ 99.9%, specification 100 mg/vial, China Institute of Veterinary Drug Control (Beijing, China), Lot: 100189-202003.

Acetonitrile, methanol, ammonium formate, and formic acid, Liquid Chromatograph-Mass spectrometric grade, Thermo Fisher Scientific Inc. (Waltham, MA, USA). Triethylamine (TEA), aqueous ammonia, ethyl acetate, sodium sulfate, acetone, sodium carbonate, sodium bicarbonate, and concentrated hydrochloric acid, analytical grade, China National Pharmaceutical Group (Sinopharm Chemical Reagent Co., Ltd., Shanghai, China). Organic nylon syringe filters, 13 mm × 0.22 µm, Jinteng company (Tianjin Jinteng Testing Equipment Co., Ltd., Tianjin, China).

Multifuge X3R high-capacity cryogenic centrifuge (Thermo Scientific, Waltham, MA, USA). Vortex Genie 2 Programmable Mixer (Scientific Industries, Bohemia, NY, USA). BUCHI Mixer B-400 tissue homogenizer (Buchi, Switzerland). AutoEVA-60 Automated Parallel Concentrator (Reeko Instrument, Xiamen, China). KQ-600DE Numerical Control Ultrasonic Cleaner (Kunshan Ultrasonic Instrument Co., Ltd., Kunshan, China). HH-S4 Thermostatic Water Bath (Zhengzhou Great Wall Technology, Zhengzhou, China).

HPLC–MS/MS with electrospray ionization (ESI) source is from the ExionAD system (AB SCIEX Corp., Framingham, MA, USA), and the Triple Quad™ 3500 mass spectrometer, data acquisition, and processing software are from OS Analyst (Version: 1.7.0.36606) (AB SCIEX Corp., Framingham, MA, USA).

### 4.2. Solution Preparation

#### 4.2.1. Standard Solution

Standard stock solution (1 mg/mL): Ten milligrams (10.00 mg) of each standard (OXY, Dr. Ehrenstorfer GmbH, Augsburg, Germany, and LEV, China Institute of Veterinary Drug Control, Beijing, China) were accurately weighed and transferred into separate 10 mL amber volumetric flasks. Each standard was dissolved and diluted to the mark with methanol to prepare stock solutions with a 1 mg/mL concentration, which were stored at −20 °C.

Serial mixed standard working solutions: A proper amount of mixed standard stock solution was accurately removed and diluted with mass spectral purity acetonitrile (Thermo Fisher Scientific Inc., Waltham, MA, USA) to prepare a serial mixed standard working solution with concentrations of 100 µg/mL, 50 µg/mL, 10 µg/mL, 5 µg/mL, 1 µg/mL, 500 ng/mL, 100 ng/mL, 50 ng/mL, and 10 ng/mL. All working solutions remained stable for up to one month when stored at −20 °C.

Internal standard stock solution (200 µg/mL): NIC (Dr. Ehrenstorfer GmbH, Augsburg, Germany) and MED (China Institute of Veterinary Drug Control, Beijing, China) were dissolved by mass spectral purity acetonitrile, mixed, and diluted to the scale, respectively, and stored at −20 °C.

#### 4.2.2. Other Solution

Acetonitrile–acetone mixed solution (6:4, *v/v*): 600 mL acetonitrile and 400 mL acetone were mixed. A 0.1 mol/L hydrochloric acid solution: 9 mL hydrochloric acid was taken into a 1000 mL volumetric flask and diluted with water to scale. A 1% triethylamine acetonitrile solution: 1 mL triethylamine was taken into a 100 mL volumetric flask and diluted with acetonitrile to scale. A 25% ammoniated acetonitrile solution: 25 mL ammonia was taken into a 100 mL volumetric flask and diluted with acetonitrile to scale. A 5% formic acid acetonitrile solution: 5 mL formic acid was taken into a 100 mL volumetric flask and diluted with acetonitrile to scale. A 4% ammoniated methanol: 4 mL ammonia was taken into a 100 mL volumetric flask and diluted with methanol to scale. Carbonate buffer: 90 mL saturated sodium bicarbonate solution and 10 mL saturated sodium carbonate solution were mixed. A 0.1% formic acid–10 mmol/L ammonium formate solution: 1 mL formic acid and 0.635 g ammonium formate were taken into a 1000 mL volumetric flask and diluted with water to scale.

### 4.3. Preparation of Sample

This study was conducted under protocols approved by the Ethics Committee of the Lanzhou Institute of Animal Husbandry and Pharmaceutical Sciences (LIAHDS-CAAS), Chinese Academy of Agricultural Sciences. All experimental procedures strictly adhered to the Implementation Guidelines for Laboratory Animal Management in Gansu Province (2005 Revision). Twenty-four clinically healthy Ovis aries (body mass range: 30–60 kg; sex ratio: 1:1) were randomly assigned into six experimental cohorts (*n* = 4 per group) using a computerized randomization protocol, maintaining equivalent gender distribution (2 males and 2 females per group).

During the 15-day acclimatization period preceding pharmaceutical intervention, all subjects received ad libitum access to standardized feed and filtered water (provisioned by Linxia Yongjing Ruilin Technology Breeding Co., Ltd., Linxia Hui Autonomous Prefecture, Gansu Province, Linxia, China), with strict exclusion of pharmacological additives. Following this stabilization phase, experimental groups received a single oral administration of compound oxyclozanide (30 g/500 mL) and levamisole hydrochloride (15 g/500 mL) suspension at therapeutic dosage levels.

Euthanasia and tissue collection were performed sequentially according to predetermined pharmacokinetic time points: Groups 1–6 underwent scheduled euthanasia at predetermined intervals: pre-administration (0 h baseline) and post-administration time points (12 h, 120 h, 264 h, 456 h, and 648 h). All surgical procedures were conducted in situ at the AAALAC-accredited facility to mitigate transportation-induced cortisol fluctuations.

Post-mortem sampling systematically collected bilateral musculus longissimus dorsi, renal tissue, hepatic parenchyma, and perirenal adipose from each specimen. Tissue samples were immediately homogenized using cryogenic pulverization (BUCHI Mixer B-400, BUCHI Labortechnik AG, St. Gallen, Switzerland), vortex-mixed for 120 s to ensure homogeneity, and aliquoted into 2 g subsamples for cryopreservation at −40 °C pending chromatographic analysis.

#### 4.3.1. Tissue Extraction from Liver, Kidney, and Muscles

Extraction of OXY and NIC: Liver, kidney, or muscle samples (2.00 ± 0.02 g) were precisely weighed and transferred into 50 mL screw-cap centrifuge tubes. A 15 mL mixture of acetonitrile–acetone (1:1, *v/v*) was added, followed by vortex mixing (1 min) and ultrasonication (15 min) to homogenize the matrix. The homogenate was agitated on a horizontal shaker (300 rpm, 10 min) and centrifuged (4500× *g*, 18 min, 4 °C). The supernatant was collected into a fresh 50 mL centrifuge tube, supplemented with 1 mL of 5% (*v/v*) ammonia solution, and stored at 4 °C pending purification.

Extraction of LEV and MED: Tissue samples (2.00 ± 0.02 g) were homogenized in 50 mL centrifuge tubes containing 2 g anhydrous sodium sulfate. After sequential addition of 1 mL carbonate buffer (pH = 9.6) and 15 mL ethyl acetate, the mixture was vortexed (2 min) and ultrasonicated (10 min) to enhance analyte partitioning. Following centrifugation (4500× *g*, 10 min, 4 °C), the supernatant was decanted, and the extraction was repeated to maximize recovery. Combined supernatants were acidified with 10 mL 0.1 mol/L HCl in a 100 mL separatory funnel. After phase stratification, the acidic layer was collected, and the extraction was repeated once to ensure quantitative transfer of target analytes.

#### 4.3.2. Purification of Tissue Extracts

Solid-Phase Extraction (SPE) for OXY and NIC: CNWBOND MAX SPE cartridges (3 mL/60 mg, CNW Technologies GmbH, Shanghai, China) were conditioned with 3 mL 25% (*v/v*) ammoniated acetonitrile. The ammonia-supplemented supernatant was loaded at a 1 mL/min flow rate. Post-loading, cartridges were sequentially washed with 3 mL ultrapure water and 3 mL methanol. After drying under reduced pressure (2.0 kPa, 5 min), analytes were eluted with 5% (*v/v*) formic acid in acetonitrile. Eluates were concentrated to dryness under nitrogen (40 °C) and reconstituted in 1.0 mL mass spectrometry-grade methanol, followed by vortex mixing (3 min) to ensure complete dissolution.

SPE for LEV and MED: Oasis MCX cartridges (3 mL/60 mg, Waters Corporation, Milford, MA, USA) were preconditioned with 3 mL methanol and 3 mL 0.1 mol/L HCl. Acidic extracts were loaded onto columns, followed by sequential washing with 3 mL water and 3 mL methanol. Cartridges were dried (2.0 kPa, 5 min) and eluted with 3 mL 4% (*v/v*) ammoniated methanol. Eluates were evaporated under nitrogen (40 °C), redissolved in 1.0 mL methanol (MS-grade), and vortexed (3 min) to homogenize residues.

All processed extracts were pooled into 5 mL centrifuge tubes, homogenized, and filtered through 0.22 μm organic-phase filter membranes prior to HPLC–MS/MS analysis.

#### 4.3.3. Perirenal Adipose Tissue Extraction

Perirenal adipose tissue samples (2.00 ± 0.02 g) were precisely weighed and transferred into 50 mL screw-cap centrifuge tubes. A 15 mL aliquot of 1% (*v/v*) triethylamine in acetonitrile solution was added, followed by vortex mixing for 1 min and ultrasonication for 15 min to achieve thorough homogenization. The homogenate was subsequently agitated on a horizontal shaker at 300 rpm for 10 min and centrifuged at 4500× *g* for 18 min at 4 °C. The supernatant was carefully decanted into a fresh 50 mL centrifuge tube, supplemented with 2 mL of 5% (*v/v*) ammonia solution, and incubated at −20 °C for 1 h to precipitate interfering substances. Following refrigeration, the mixture was recentrifuged under identical conditions (4500× *g*, 18 min, 4 °C), and the clarified supernatant was collected for subsequent purification.

For the extraction of levofloxacin (LEV) and metronidazole (MED) from Perirenal adipose, the protocol described in Section 4.3.1 for liver, kidney, and muscle tissues was strictly replicated to ensure methodological consistency.

#### 4.3.4. Perirenal Adipose Tissue Purification

The purification of Perirenal adipose extracts followed the SPE protocol outlined in Section 4.3.2 for hepatic, renal, and muscle tissues. Briefly, CNWBOND MAX SPE cartridges (3 mL/60 mg, CNW Technologies GmbH, Shanghai, China) or Oasis MCX cartridges (3 mL/60 mg, Waters Corporation) were employed for target analyte isolation, depending on the compound class (OXY/NIC vs. LEV/MED). Cartridge conditioning, sample loading, washing, drying, and elution steps were executed as previously described, ensuring uniformity in solvent volumes (3 mL per step), flow rates (1 mL/min), and elution conditions (5% formic acid in acetonitrile or 4% ammoniated methanol). Eluates were evaporated to dryness under nitrogen at 40 °C, reconstituted in 1.0 mL mass spectrometry-grade methanol, and vortexed for 3 min to ensure complete dissolution prior to HPLC–MS/MS analysis.

### 4.4. HPLC–MS/MS Instruments, Methods, and Conditions

A mass spectrometer (AB SCIEX Corp., Framingham, MA, USA) with an electrospray ionization source interface operated in the positive ion electrospray ionization (ESI+)/negative ion electrospray ionization (ESI−) of multiple reaction monitoring (MRM) mode was used for the HPLC–MS/MS (AB SCIEX Corp., Framingham, MA, USA) analysis. The internal standard method was used for quantification by a calibration curve of standard solutions.

#### 4.4.1. Chromatographic Condition

Waters ACQUITY PRM BEH C_18_: 1.7 μm, 2.1 × 100 mm. Mobile phase A was 0.1% formic acid-10 mmol/L ammonium formate solution, and phase B was acetonitrile. Column temperature, 35 °C; injection volume, 5 µL; flow rate, 0.3 mL/min; injector temperature, 5 °C.

#### 4.4.2. Mass Spectrometer Conditions

Electrospray ion source (ESI) with ESI+ and ESI− ionization mode: Quantitative detection was performed in the multi-ion reaction monitoring (MRM) mode. In ESI+ mode, capillary voltage is set to + 5500 V; in ESI− mode, Capillary voltage is −4500 V. Ion source temperature, 550 °C; curtain gas (N_2_), 35 psi; ion source spray gas (N_2_), 55 psi; auxiliary heating gas (N_2_), 5 psi.

### 4.5. Investigation of Method

The validation of this method was conducted in accordance with General Rule 9012 of the Pharmacopoeia of the People’s Republic of China (PRC) titled “Verification of Quantitative Analysis Methods for Biological Samples”, and the International Cooperation on Harmonisation of Technical Requirements for the Registration of Veterinary Medicinal Products (VICH) guideline entitled “Studies to Evaluate the Metabolism and Residue Kinetics of Veterinary Drugs in Food-producing Animals: Validation of Analytical Methods Used in Residue Depletion Studies”.

The validation of this analytical method was performed in compliance with the following international standards: (i) General Chapter 9012 (“Verification of Quantitative Analysis Methods for Biological Samples”) from The Pharmacopoeia of the People’s Republic of China [36]; (ii) the International Council for Harmonisation of Technical Requirements for Pharmaceuticals for Human Use (ICH) guideline Q2(R1), “Validation of Analytical Procedures: Text and Methodology [37]; and (iii) the International Cooperation on Harmonisation of Technical Requirements for the Registration of Veterinary Medicinal Products (VICH) guideline, “Studies to Evaluate the Metabolism and Residue Kinetics of Veterinary Drugs in Food-producing Animals: Validation of Analytical Methods Used in Residue Depletion Studies [38]. The decision limit calculation was performed per the Decision 2002/657/EC [39]. The calculation of the withdrawal period was conducted in accordance with the Guidelines for the Elimination of Veterinary Drug Residues (No. 326) issued by the Ministry of Agriculture and Rural Affairs of the People’s Republic of China (MARA) [40].

#### 4.5.1. Investigation of Specificity

The specificity of the method was assessed using a mixed standard solution, a blank matrix, and a blank matrix spiked with only one internal standard and test sample (15 μg/kg).

#### 4.5.2. Investigation of Sensitivity

The limit of detection (LOD) and limit of quantitation (LOQ) were determined by analyzing each standard concentration seven times. The LOD was defined as the minimum signal-to-noise ratio (S/N) ratio achieving ≥3 for the target ions, while LOQ was established at an S/N ratio of ≥10, ensuring reliable quantification capacity [41].

#### 4.5.3. Investigation of Matrix Effects

Matrix factors for analytes and internal standards were determined through post-spiking experiments by comparing their responses in the test matrix versus solvent. Matrix effects were quantified using internal standard-corrected response ratios (post-spiking/extraction), where signal suppression/enhancement was normalized through IS-based quantification [42].

#### 4.5.4. Investigation of Linearity

The mixed standard solutions of OXY and LEV were prepared with the concentrations of 2.5 ng/mL, 5 ng/mL, 8 ng/mL, 10 ng/mL, 15 ng/mL, 20 ng/mL, 25 ng/mL, 30 ng/mL, 40 ng/mL, 50 ng/mL, 100 ng/mL, 200 ng/mL, and 250 ng/mL, and the concentrations of NIC and MED were 20 ng/mL. Standard curves were generated by plotting the normalized response ratios (OXY/LEV: IS) against the corresponding concentration ratios (OXY/LEV: IS) using logarithmic coordinates. A weighted least-squares regression model with 1/x^2^ weighting was employed to optimize the linear relationship, and the resultant equations were derived to establish calibration parameters.

#### 4.5.5. Investigation of Accuracy and Precision

Blank matrix test materials containing OXY and LEV at concentrations of 2.5, 5, 10, 20, 50, 100, 200, 250, 500, and 1000 µg/kg were prepared for different tissue matrices. The internal standards (NIC and MED) were added at a constant concentration of 20 µg/kg across all matrices. Six replicate samples were prepared at each concentration level to ensure experimental robustness. Using the validated analytical procedure, the method’s accuracy, within-batch precision, and intermediate precision were systematically evaluated in accordance with VICH GL 49(R) [38] and ICH Q2(R1) [37] guidelines.

#### 4.5.6. Stability in Matrix and Process Sample Stability

Target analytes were spiked into various tissue matrices at predetermined concentrations in accordance with VICH GL 49(R) and ICH Q2(R1) guidelines to evaluate stability under three experimental conditions: short-term stability evaluation at ambient temperature (25 ± 2 °C) for 8 h; RSD assessment through three sequential freeze-thaw cycles (−20 °C to 4 °C); and long-term stability monitoring at −70 °C over a 3-month period.

### 4.6. Investigation of Withdraw Period

Following the prescribed method, tissues were collected at designated time points, pretreated, and analyzed by HPLC-MS. Based on the test results, withdrawal periods were determined via non-linear regression analysis using the EMA-authorized WTM1.4 software.

## 5. Conclusions

This study successfully developed a highly sensitive and reliable HPLC–MS/MS method for the simultaneous determination of LEV and OXY in ovine tissues (liver, perirenal adipose, kidneys, and muscles). The optimized protocol employs structural analogues as internal standards for quantitative analysis, achieving a limit of detection (LOD) below 2 μg/kg for both analytes. Validation demonstrated excellent recoveries (>80.00%) with minimal matrix interference, fully complying with EU regulatory requirements for confirmatory analysis. Application of this method to 24 post-dosing sheep samples confirmed its robustness, specificity, and practicality. Notably, the technique successfully identified both LEV and OXY residues in tissue matrices following administration of the veterinary compound oxyclozanide–levamisole suspension. These findings provide critical methodological validation and pharmacokinetic evidence supporting the clinical efficacy of this drug combination, establish a standardized analytical framework for residue monitoring in livestock, and offer an essential technical reference for veterinary drug development and food safety control.

## Figures and Tables

**Table 1:** Gradient elution program of liquid chromatography.

Time (min)	Mobile Phase A (%)	Mobile Phase B (%)
0	90	10
1	90	10
2	52	48
6	52	48
8	20	80
10	20	80
11	90	10
13	90	10

**Table 2:** Qualitative and quantitative ion pairs and declustering potential and collision energy values for OXY and LEV.

Sample	Polarity	Precursor Ion (*m*/*z*)	Product Ion (*m*/*z*)	DP (V)	CE (eV)	Retention Time
LEV	+	205.1	123.0 */178.1	140	38/29	2.94
MED	+	296.1	264.1 */104.8	150	30/46	3.83
OXY	−	399.8	203.9 */196.7	−80	−33/−37	8.54
NIC	−	325.1	288.8 */170.9	−118	−24/−36	8.67

Note: LEV: levamisole hydrochloride; MED: mebendazole; OXY: oxyclozanide; NIC: niclosamide; DP: declustering potential; CE: collision energy; *: quantitative ion.

**Table 3:** The matrix effects of LEV and OXY in liver tissue (perirenal adipose, kidney, and muscles) (%).

Tissue	Concentration	OXY (%)	LEV (%)
Liver	LOQ	13.63	9.36
	2MRL	12.55	8.07
Perirenal adipose	LOQ	14.28	11.91
	2MRL	9.98	7.38
Kidney	LOQ	13.43	14.80
	2MRL	12.05	12.27
Muscles	LOQ	12.95	13.47
	2MRL	11.03	3.61

Note: Matrix effects are expressed as relative bias (%) calculated against the reference method.

**Table 4:** Typical calibration curve equation of each analyte.

Tissue	Analyte	Regression Equation	Determination Coefficient (R^2^)	Linearity Range (μg/kg)
Liver	LEV	y = 0.10099 x − 0.00337	0.99296	2.50–250.00
	OXY	y = 0.11969 x + 0.31266	0.99733	
Perirenal adipose	LEV	y = 0.01764 x + 0.02004	0.99753	2.50–50.00
	OXY	y = 0.02375 x − 0.03083	0.99409	
Kidney	LEV	y = 0.07637 x − 0.17145	0.99752	2.50–100.00
	OXY	y = 0.34327 x − 0.55890	0.99571	
Muscles	LEV	y = 0.01084 x + 0.00683	0.99476	2.50–50.00
	OXY	y = 0.01963 x − 0.02379	0.99273	

**Table 5:** Accuracy and precision of LEV and OXY added to blank tissue (*n* = 7).

Tissue	Analyte	Addition Level (μg/kg)	Recovery (%)	Intraday RSD (%)	Inter-Day RSD (%)
Liver	LEV	2.50	108.62 ± 5.63	5.34	7.05
		50.00	89.39 ± 2.63	2.96	8.74
		100.00	91.19 ± 3.72	4.12	4.45
		200.00	95.30 ± 1.64	1.73	3.54
	OXY	2.50	115.36 ± 3.13	2.72	1.21
		250.00	90.47 ± 1.47	1.71	13.43
		500.00	101.49 ± 1.47	1.44	4.16
		1000.00	97.74 ± 4.29	4.63	12.63
Perirenal adipose	LEV	2.50	86.29 ± 8.92	10.62	8.45
		5.00	100.40 ± 6.49	6.46	8.66
		10.00	103.19 ± 4.71	4.62	3.54
		20.00	91.99 ± 5.79	6.29	2.72
	OXY	2.50	87.98 ± 11.04	12.51	13.75
		10.00	94.40 ± 4.88	5.24	5.42
		20.00	88.45 ± 4.66	5.99	8.25
		40.00	91.68 ± 5.86	6.28	6.31
Kidney	LEV	2.50	113.13 ± 5.66	5.12	4.57
		5.00	100.46 ± 6.77	6.68	2.68
		10.00	87.61 ± 3.18	3.61	5.48
		20.00	86.73 ± 2.52	2.94	5.88
	OXY	2.50	96.75 ± 10.70	11.03	4.96
		50.00	88.63 ± 3.28	3.59	15.58
		100.00	100.68 ± 4.14	4.13	6.41
		200.00	102.33 ± 3.72	3.75	5.03
Muscles	LEV	2.50	87.40 ± 7.67	9.55	17.70
		5.00	97.53 ± 5.57	5.67	9.11
		10.00	94.33 ± 5.66	5.95	3.24
		20.00	86.95 ± 4.26	4.85	4.61
	OXY	2.50	87.25 ± 8.87	10.51	0.29
		10.00	95.36 ± 8.08	8.46	2.81
		20.00	80.94 ± 5.85	7.25	7.27
		40.00	92.28 ± 3.85	4.19	2.89

## Data Availability

The data that support the findings of this study are available from the corresponding author upon reasonable request. Some data may not be made available because of privacy or ethical restrictions.

## Supplementary Materials

The following supporting information can be downloaded at https://www.mdpi.com/article/10.3390/molecules30071473/s1: Figure S1. The scan chromatogram of each compound. a-LEV, b-MED, c-OXY, d-NIC; Figure S2. TIC and MRM chromatogram. A. TIC B. LEV1 205.1 > 123.0 *. C. MED1 296.1 > 264.1 *. D. NIC1 325.1 > 288.8 *. E. XY 399.8 > 203.9 *; Figure S3. Blank matrix MRM chromatogram. A. Perirenal adipose-TIC. B. Perirenal adipose-LEV, MED. C. Perirenal adipose-OXY, NIC. D. Kidney-TIC. E. Kidney-LEV, MED. F. Kidney-OXY, NIC. G. Liver-TIC. H. Liver-LEV, MED. I. Liver-OXY, NIC. J. Muscle-TIC. K. Muscle-LEV, MED. L. Muscle-OXY, NIC; Figure S4. Blank matrix with internal standard MRM chromatogram. A. Muscle-TIC. B. Muscle-MED. C. Muscle-LEV. D. Muscle-NIC. E. Muscle-OXY. F. Liver-TIC. G. Liver-MED. H. Liver-LEV. I. Liver-NIC. J. Liver-OXY. K. Kidney-TIC. L. Kidney-MED. M. Kidney-LEV. N. Kidney-NIC. O. Kidney-OXY. P. Perirenal adipose-TIC. Q. Perirenal adipose-MED. R. Perirenal adipose-LEV. S. Perirenal adipose-NIC. T. Perirenal adipose-OXY; Table S1. Concentrations and RSD of LEV and OXY in various tissues after 8 hours at room temperature (*n* = 7); Table S2. Concentrations and RSD of LEV and OXY in various tissues after three cycles of freeze-thawing (*n* = 7); and Table S3. Concentrations and RSD of LEV and OXY in various tissues after 1, 2, and 3 months of cryopreservation (*n* = 7).
