# Supplementary material for: Dual SPE–HPLC–MS/MS Platform for Cross-Class Antiparasitic Surveillance: Simultaneous Quantification of Oxyclozanide and Levamisole Hydrochloride in Ovine Tissues with Applications to Withdrawal Period Optimization
Source: Molecules. 2025 Mar 26;30(7):1473. doi: 10.3390/molecules30071473 (PMC11990247; doi:10.3390/molecules30071473)
Supplement: Supplementary file 1 [file molecules-30-01473-s001.zip › molecules-3535345-supplementary.pdf]

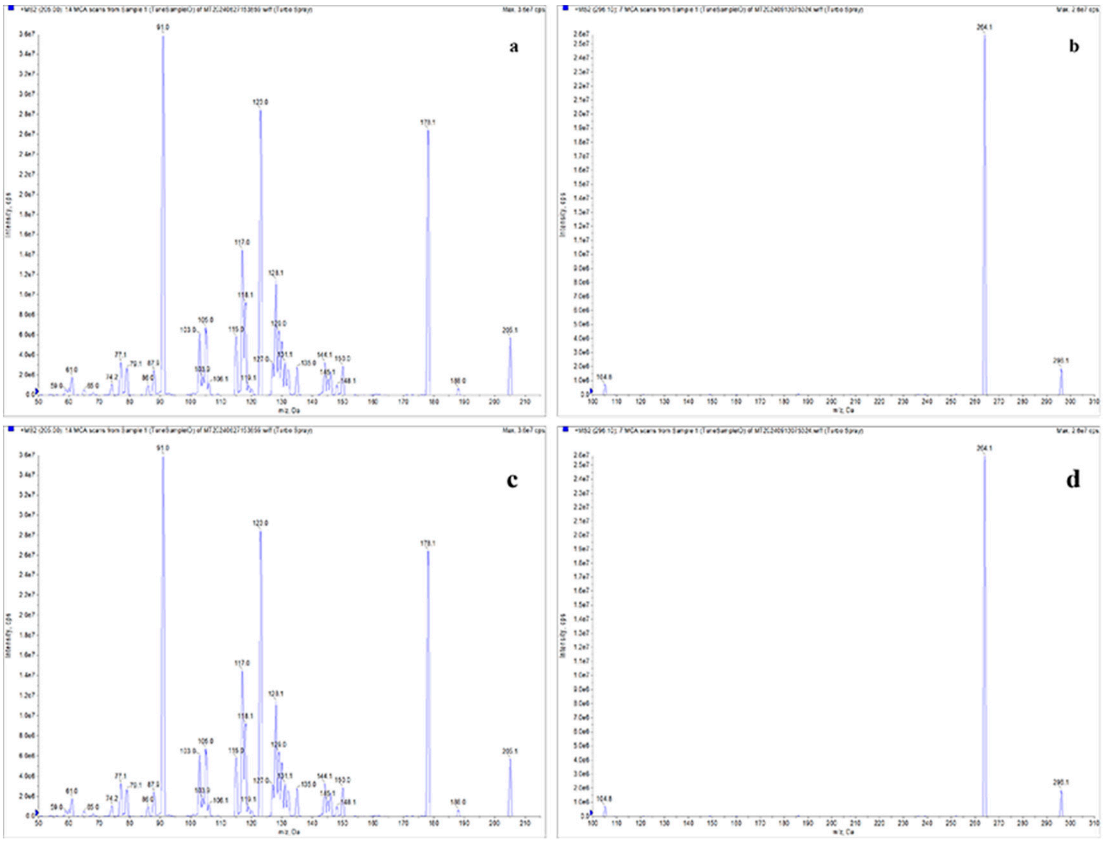


**Figure S1.** The scan chromatogram of each compound. a-LEV, b-MED, c-OXY, d-NIC

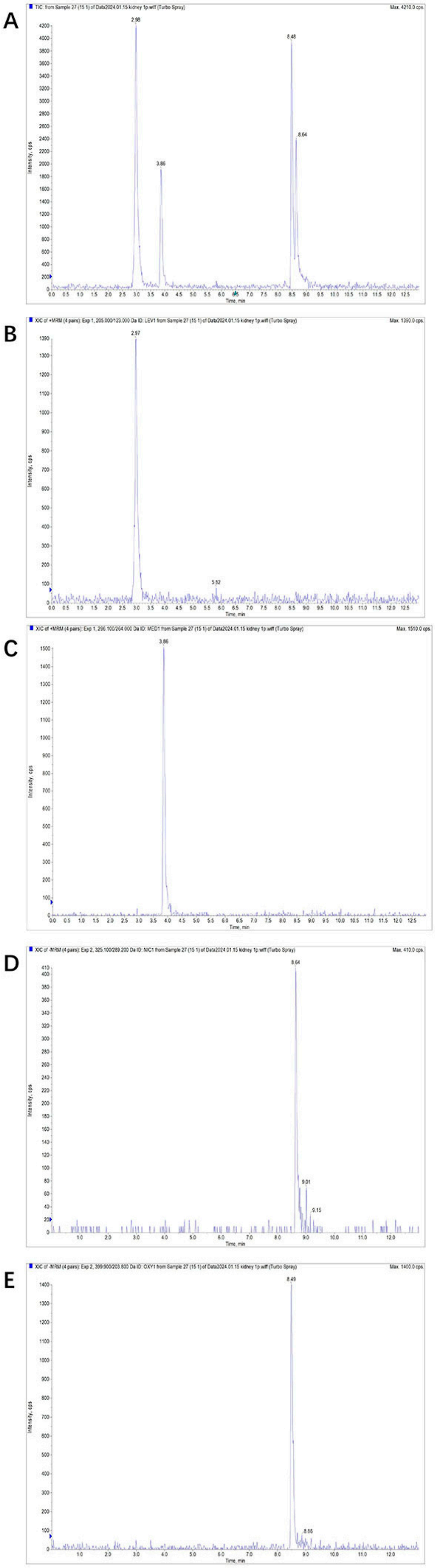
A
TIC: from Sample 27 (15 1) of Data2024.01.15 kidney 1p.wiff (Turbo Spray)
Max. 4210.0 cps.
2.98
3.86
8.48
8.64
Intensity, cps
Time, min
B
XIC of +MRM (4 pairs): Exp 1, 205.000/123.000 Da ID: LEV1 from Sample 27 (15 1) of Data2024.01.15 kidney 1p.wiff (Turbo Spray)
Max. 1390.0 cps.
2.97
5.82
Intensity, cps
Time, min
C
XIC of +MRM (4 pairs): Exp 1, 296.100/264.000 Da ID: MED1 from Sample 27 (15 1) of Data2024.01.15 kidney 1p.wiff (Turbo Spray)
Max. 1510.0 cps.
3.86
Intensity, cps
Time, min
D
XIC of -MRM (4 pairs): Exp 2, 325.100/289.200 Da ID: NIC1 from Sample 27 (15 1) of Data2024.01.15 kidney 1p.wiff (Turbo Spray)
Max. 410.0 cps.
8.64
9.01
9.15
Intensity, cps
Time, min
E
XIC of -MRM (4 pairs): Exp 2, 399.900/203.800 Da ID: OXY1 from Sample 27 (15 1) of Data2024.01.15 kidney 1p.wiff (Turbo Spray)
Max. 1400.0 cps.
8.49
8.86
Intensity, cps
Time, min

**Figure S2**. TIC and MRM chromatogram. A. TIC B. LEV1 205.1>123.0*. C. MED1 296.1>264.1*. D. NIC1 325.1>288.8*. E. OXY1 399.8>203.9*.

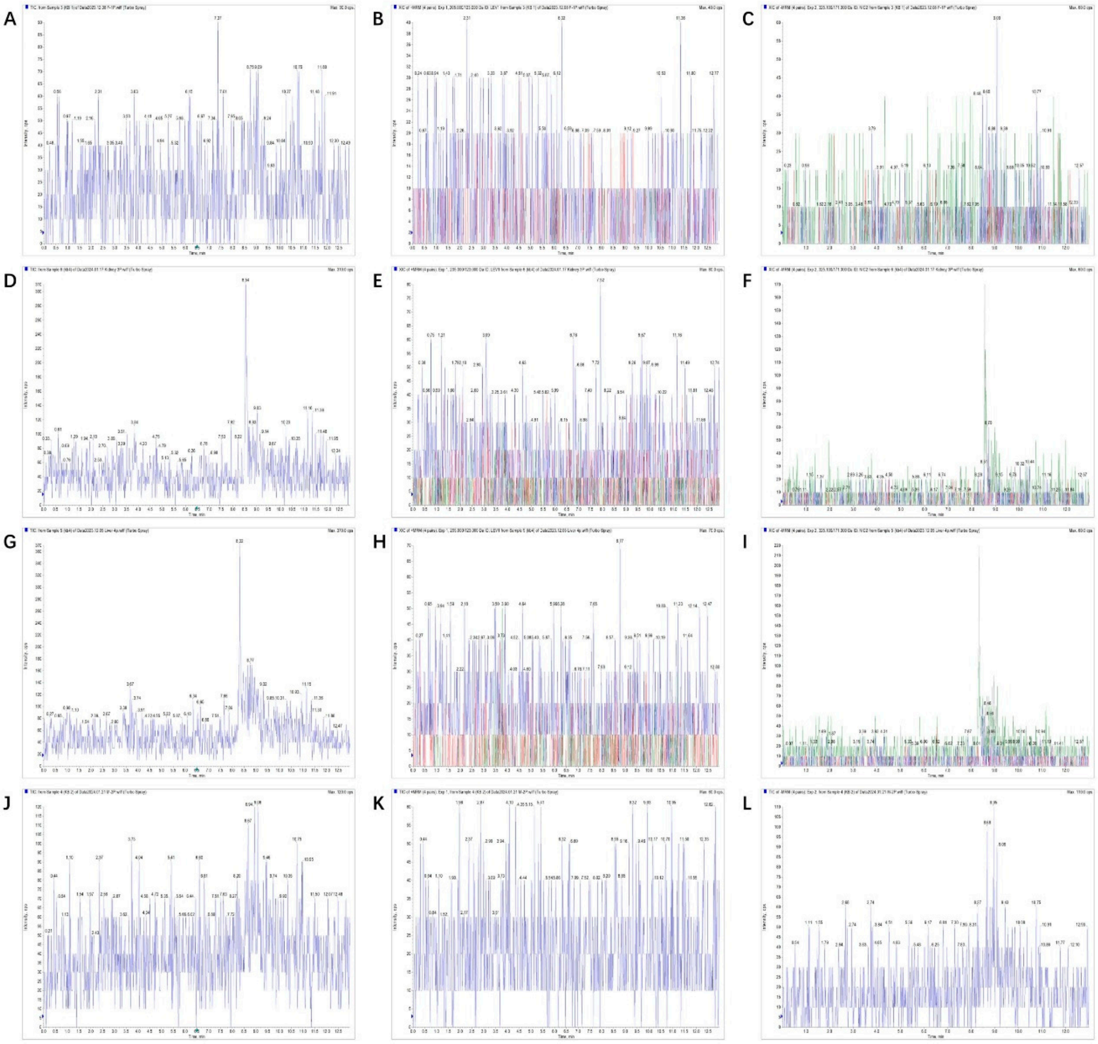


**Figure S3.** Blank matrix MRM chromatogram.

A. Perirenal adipose-TIC. B. Perirenal adipose-LEV, MED. C. Perirenal adipose-OXY, NIC.

D. Kidney-TIC. E. Kidney-LEV, MED. F. Kidney-OXY, NIC.

G. Liver-TIC. H. Liver-LEV, MED. I. Liver-OXY, NIC.

J. Muscle-TIC. K. Muscle-LEV, MED. L. Muscle-OXY, NIC.

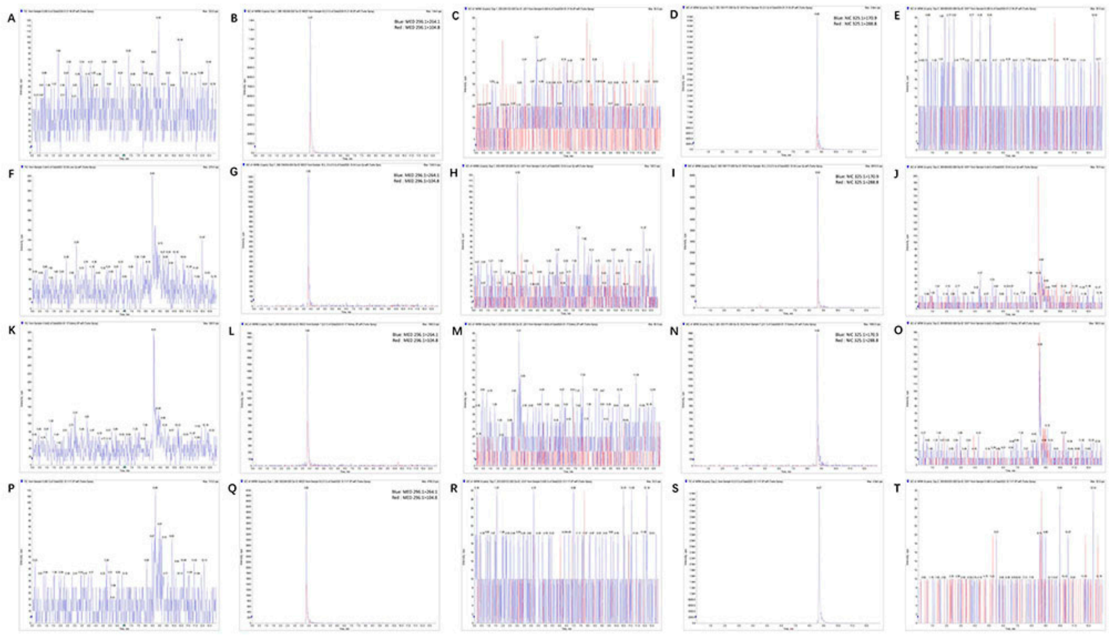

**Figure S4.** Blank matrix with internal standard MRM chromatogram.

A. Muscle-TIC. B. Muscle-MED. C. Muscle-LEV. D. Muscle-NIC. E. Muscle-OXY.

F. Liver-TIC. G. Liver-MED. H. Liver-LEV. I. Liver-NIC. J. Liver-OXY.

K. Kidney-TIC. L. Kidney-MED. M. Kidney-LEV. N. Kidney-NIC. O. Kidney-OXY.

P. Perirenal adipose-TIC. Q. Perirenal adipose-MED. R. Perirenal adipose-LEV. S. Perirenal adipose-NIC. T. Perirenal adipose-OXY.

Table S1. Concentrations and RSD of LEV and OXY in various tissues after 8 hours at room temperature (n = 7)

| Tissue | Analyte | Addition Level (μg/kg) | Concentration (μg/kg) | Recovery (%) | Intraday RSD (%) |
|---|---|---|---|---|---|
| Liver | LEV | 2.50 | 1.95±0.19 | 77.96±7.62 | 9.83 |
| | | 200.00 | 181.65±3.24 | 90.83±1.62 | 1.82 |
| | OXY | 2.50 | 2.37±0.29 | 94.62±11.63 | 12.29 |
| | | 1000.00 | 907.87±22.62 | 90.79±2.26 | 2.49 |
| Perirenal adipose | LEV | 2.50 | 2.30±0.18 | 92.03±7.21 | 8.04 |
| | | 20.00 | 19.24±0.51 | 96.20±2.57 | 2.68 |
| | OXY | 2.50 | 2.18±0.31 | 87.17±12.59 | 14.40 |
| | | 40.00 | 37.14±1.68 | 92.84±4.20 | 4.57 |
| Kidney | LEV | 2.50 | 2.62±0.11 | 104.98±4.39 | 4.17 |
| | | 20.00 | 17.85±0.57 | 89.27±2.84 | 3.18 |
| | OXY | 2.50 | 2.80±0.14 | 111.97±5.49 | 4.90 |
| | | 200.00 | 202.08±4.21 | 101.04±2.11 | 2.14 |
| Muscles | LEV | 2.50 | 2.87±0.10 | 114.64±3.84 | 3.35 |
| | | 20.00 | 18.95±0.60 | 94.75±3.01 | 3.13 |
| | OXY | 2.50 | 1.88±0.18 | 75.11±7.08 | 9.66 |
| | | 40.00 | 39.19±1.54 | 97.98±3.84 | 3.89 |

Table S2. Concentrations and RSD of LEV and OXY in various tissues after three cycles of freeze-thawing (n = 7)

| Tissue | Analyte | Addition Level (μg/kg) | Concentration (μg/kg) | Recovery (%) | Intraday RSD (%) |
|---|---|---|---|---|---|
| Liver | LEV | 2.50 | 1.95±0.16 | 77.96±6.20 | 7.96 |
| | | 200.00 | 179.98±2.88 | 89.99±1.44 | 1.58 |
| | OXY | 2.50 | 2.42±0.21 | 96.92±8.33 | 8.57 |
| | | 1000.00 | 888.67±55.02 | 88.87±5.50 | 6.16 |
| Perirenal adipose | LEV | 2.50 | 2.29±0.21 | 91.79±8.32 | 9.20 |
| | | 20.00 | 19.39±0.33 | 96.94±1.65 | 1.70 |
| | OXY | 2.50 | 2.27±0.29 | 90.65±11.54 | 12.72 |
| | | 40.00 | 40.92±1.07 | 102.30±2.68 | 2.62 |
| Kidney | LEV | 2.50 | 2.79±0.10 | 111.74±3.86 | 3.45 |
| | | 20.00 | 17.43±0.73 | 87.15±3.63 | 4.17 |
| | OXY | 2.50 | 2.87±0.14 | 114.64±5.45 | 4.75 |
| | | 200.00 | 209.54±2.85 | 104.77±1.43 | 1.36 |
| Muscles | LEV | 2.50 | 2.89±0.09 | 115.42±3.49 | 3.02 |
| | | 20.00 | 19.54±0.60 | 97.70±3.02 | 3.08 |
| | OXY | 2.50 | 1.96±0.28 | 78.48±11.39 | 14.48 |
| | | 40.00 | 38.92±0.85 | 97.31±2.12 | 2.18 |

Table S3. Concentrations and RSD of LEV and OXY in various tissues after 1, 2, and 3 months of cryopreservation (n = 7)

| Tissue | Analyte | Addition Level (μg/kg) | Concentration (μg/kg) | Recovery (%) | Intraday RSD (%) |
|---|---|---|---|---|---|
| Liver | LEV | 2.50 | 2.84±0.13 | 113.77±5.21 | 4.60 |
| | | 200.00 | 191.57±5.84 | 99.61±2.92 | 3.06 |
| | OXY | 2.50 | 2.92±0.05 | 116.93±1.96 | 1.69 |
| | | 1000.00 | 1027.07±49.97 | 102.71±5.00 | 4.90 |
| Perirenal adipose | LEV | 2.50 | 2.12±0.21 | 84.84±8.43 | 9.82 |
| | | 20.00 | 19.34±0.83 | 96.68±4.14 | 4.29 |
| | OXY | 2.50 | 20.21±0.21 | 95.46±8.24 | 8.64 |
| | | 40.00 | 41.57±0.67 | 103.92±1.66 | 1.60 |
| Kidney | LEV | 2.50 | 2.68±0.18 | 107.06±7.23 | 6.69 |
| | | 20.00 | 18.46±0.70 | 92.28±3.52 | 3.86 |
| | OXY | 2.50 | 2.86±0.10 | 114.41±3.85 | 3.38 |
| | | 200.00 | 209.50±3.44 | 104.75±1.72 | 1.64 |
| Muscles | LEV | 2.50 | 2.41±0.23 | 96.24±9.21 | 9.70 |
| | | 20.00 | 17.32±0.81 | 86.61±4.07 | 4.49 |
| | OXY | 2.50 | 2.34±0.30 | 93.76±10.60 | 12.57 |
| | | 40.00 | 38.79±1.15 | 96.97±3.24 | 4.80 |
